# Investigation of the Reusability of a Polyurethane-Bound Noise-Absorbing Pavement in Terms of Reclaimed Asphalt Pavement

**DOI:** 10.3390/ma15093040

**Published:** 2022-04-22

**Authors:** Sabine Faßbender, Markus Oeser

**Affiliations:** Institute of Highway Engineering, RWTH Aachen University, Mies-van-der-Rohe-Str. 1, 52074 Aachen, Germany; info@isac.rwth-aachen.de

**Keywords:** noise reduction, recycled material, polyurethane-bound road layer, mechanical performance, low temperature, deformation, fatigue, polyurethane-based reclaimed asphalt pavement (PU-RAP)

## Abstract

A key aspect of sustainable pavement construction is the use of environmentally-friendly designed pavement materials. These materials are characterized by the fact that they are renewable raw materials, require a low amount of energy during production and in the best case, are made from a high proportion of recyclable materials in order to reduce waste. A number of recent studies have demonstrated the recyclability of waste materials that can be very well utilized in road construction. This study describes the recycling of a new and innovative topcoat system that already contains recycled materials. However, the focus is on guaranteeing the mechanical performance of the innovative absorption layer where different portions of used material are added. Therefore, low-temperature behaviour, durability, fatigue and noise absorption are investigated in detail and it is concluded that their function is preserved. In order to investigate these characteristics, the impedance measuring tube, the uniaxial cyclic compression test (UCCT), the three point bending test (3PB), the uniaxial tension stress test (UTST) and the thermal stress restrained specimen test (TSRST) are used. However, the examined absorption material can be reused to build innovative roads.

## 1. Introduction

Sustainability is one of the most important issues to protect planet Earth and to provide a good life for future generations. In the wake of this realization, action plans have been developed worldwide to protect and regenerate our planet.

The new Circular Economy Action Plan of the European Commission was adopted in 2020 [1]. This calls on the population to implement the European Green Deal, which requires the EU to promote regenerative growth by giving more back to the planet than is taken from it. In addition, resource consumption should be within the planet’s carrying capacity, leading everyone to reduce their ecological footprint. Finally, another key goal is to double the circular use of materials in the coming years [1]. It was not only the European Commission that developed an action plan. The United Nations also have a sustainable development agenda for the world. This provides 17 goals to be implemented to achieve a sustainable planet. In addition to social and health goals, the focus is also on infrastructure and consumption goals. Goals 9 and 12, for example, aim to develop a resilient infrastructure and promote sustainable consumption and products. Here, the efficient use of natural resources and waste reduction are of great importance [2].

One approach in this process is the use of sustainable materials in the construction industry. Sustainable materials are characterized by the fact that they are renewable raw materials, require a low amount of energy during production and in the best case, are additionally made from a high proportion of recyclable materials.

In the course of developing an innovative pavement, a noise reducing two layer surface was developed by Faßbender and Oeser [3] first. Here, a bottom layer, which enables the absorption of noise, is made of rock, crump rubber and polyurethane. A void-rich pore structure of the material from [3] results in a high absorption capacity, which leads to a great noise reduction. Additionally, the material delivers high mechanical strength and durability.

The study by Faßbender and Oeser is based on the international findings of Sandberg, Ejsmont, Goubert and Schacht ([4,5,6,7]) which dealt with the investigation of poro-elastic pavements (PERS). PERS is a road surface with a high void content (30–40 vol.%) made of aggregates, rubber particles (up to 90 wt.%) and polyurethane binder.

The binder polyurethane replaces bitumen in the invented mixes. Polyurethane exists as an one- or two-component system composed of a polyol and an isocyanate. During production, a polyaddition reaction of polyols and isocyanates takes place, resulting in the formation of a urethane group [8]. Polyurethane can be manufactured from a variety of available raw materials and offers a wide range of applications with high stability [9,10].

The development of high-performance road materials helps to ensure long-term durability and the relevant function of roads. To this end, many experiments on high-performance construction materials such as polyurethane-bonded asphalts have been done in the past. In particular, the research reports from the research projects LIDAK [11], INNO-BOND [12], INNO-PAVE [13] and the works from Renken [10,14], Schacht [7], Lu [15] and Faßbender and Oeser [3] provide sufficient evidence of the sustainability of polyurethane-bound and permeable asphalt pavements. They are characterized as follows:

- They use polyurethane binder (i.e., Elastopave^®^), of which 83% of the polyol component can be produced from renewable raw materials (e.g., ricinus oil) and thus, they distance themselves from the conventional binder bitumen, which is not a renewable raw material [10].
- They provide infiltration capacity of surface water and thus prevent flooding [10,15].
- They can reduce the heat island effect in cities, because of its density and low heat storage capacity [10].
- They offer a high noise reduction potential due to their cavity-rich structure with simultaneous long-term durability [3,10,15].

In addition to the development of the innovative and sustainable pavement materials, the potential of the recyclability of these materials is also of great importance. In Germany, conventional asphalt recycling is generally carried out by adding reclaimed asphalt pavement material (RAP) to new Hot Mix Asphalt. Only RAP that is suitable according to the *Technical Terms of Delivery for Asphalt Granulate TL AG-STB 2009* [16] may be used. In order to keep the quality of the milled material high, layer-by-layer milling is recommended [17].

A distinction is made in the use of asphalt granulate between recycling and reuse. Recycling describes the process by which the added asphalt is processed into a new material and is then used, for example, in construction material mixtures for base layers with hydraulic binders. Reuse is the repeated use of a material, in which RAP is added to Hot Mix Asphalt [17]. If RAP is to be used in asphalt layers, the maximum amount to be added is calculated depending on the properties of the RAP. The addition of the RAP influences the quality of the fresh Hot Mix Asphalt, which must meet the requirements of the *Technical Terms of Delivery for Asphalt TL Asphalt -StB*. Once all quality requirements have been fulfilled, the mix is paved.

Against this background, the question arises whether innovative mixed pavement mixtures such as those of Refs [3,10,15] can also certainly be reused proportionately and whether this affects the mechanical properties of the finished material.

Building on the previous study by Faßbender and Oeser [3], the current study aims to find out whether the absorbing layer can be reused and shows similar material performance. This leads to the intent of this study, which is to investigate the extent to which polyurethane-based reclaimed asphalt pavement material (PU-RAP) can be used as an additive material for new polyurethane-bound asphalt mixes and whether the use of PU-RAP imposes any limitations on the functionality of the pavement. At the same time, the comparison is drawn with the variant from [3] without recycled components.

## 2. Methodology and Materials

### 2.1. Methodology

To answer the question as to whether the substitution of aggregates by PU-RAP degrades the material properties, a clear methodology is applied. The developed material from [3] is used as the basis for this study in order to enable a concrete comparison of the material properties of the freshly composed mix and the mix produced with PU-RAP.

For this purpose, in the first step, the mix from [3] is produced and processed into PU-RAP (see Section 2.2.1). Then, the material is manufactured again, but PU-RAP is substituted in different proportions. The material properties are then determined for the different variants. This is followed by the investigation of the material performance on the specimens, which consists of the investigation of the absorption behaviour, the deformation behaviour, the fatigue behaviour and the low-temperature behaviour.

### 2.2. Material

The grading curve developed by Faßbender and Oeser [3] is used as the starting point. The grading curve distribution was developed to provide optimal mechanical and acoustic properties of the poro-elastic absorption layer. The developed grading curve distribution forms an aggregate structure with a high void content. The base aggregate material consists of 4 vol.% limestone filler, 3 vol.% basalt sand, 85.5 vol.% basalt crushed stone and 7.5 vol.% rubber granules from Genan GmbH (Dorsten, Germany) (also see Table A1). To this mix, polyurethane from BASF Polyurethanes GmbH (Lemförde, Germany) was added as binder to ensure the functionality (see Table 1) and replaces the conventionally used bitumen binder. The rubber granulate consists of recycled car tires and has grain sizes from 2 to 6 mm. In this work, the one-component binder used in the previous study [3] is replaced by a two-component polyurethane binder which is assumed to increase the final stiffness. The two-component binder is composed of a polyol (Elastopave 6551/102) and an isocyanate (IsoPMDI 92140), which are mixed before being applied to the aggregates. The mixing ratio indicated by the manufacturer is 100:68. The polyol is based on vegetable oils, 45% of which are made from renewable raw materials (see product information form from BASF Polyurethanes GmbH in [10]). Table A2 in the Appendix A shows typical physical properties of the used polyurethane Elastopave^®^ 6551/102.

#### 2.2.1. Preparation of the PU-RAP

**Figure 1:**
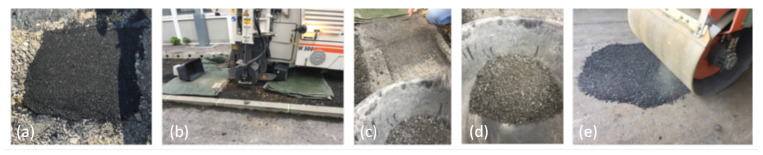
Preparation of the PU-RAP: (**a**) PU-asphalt pavement section on construction site. (**b**) Milling of the PU pavement. (**c**) View on the milled fractions after milling. (**d**) PU-RAP placed in bucket (**e**) Crushing of the PU-RAP by a roller compactor.

In order to produce the PU based reclaimed asphalt pavement (PU-RAP), approximately 280 kg of the base material was prepared in a batch mixer and backfilled in the paving section of the Institute of Highway Engineering. For this purpose, a hole with a depth of 30 cm was excavated and filled with the polyurethane mixture. After curing of the mix, the layer was broken up. This process was done after 48h with the help of a conventional asphalt milling machine (Figure 1b). The layer was milled only in the centre to avoid contamination of the milled material by the surrounding soil. After this, a roller compactor broke the material once again due to rolling over it on the ground in order to receive finer material (Figure 1e). The milled and crushed material was collected and stored dry in containers. During the production of the PU-RAP, no separation of polyurethane and aggregates was carried out, which means that the old polyurethane is present in the PU-RAP as a solid component which adheres to the aggregate. Figure 1 provides insight into the preparation process.

Furthermore, the PU-RAP was split into its individual fractions. After initial screening, it was found that the milled material had a too high oversize content to produce a grading curve according to [3] (high proportion of grain fractions larger than 8.0 mm in diameter). In order to have sufficient PU-RAP available for the production of the test variants, the oversize fraction was reduced once again by passing a compaction roller on a rigid base. The subsequent screening of the PU-RAP yielded a total mass of 80 kg in three different grain fractions. These three grain fractions occupy the range of 0–8 mm grain diameter (Figure 1e). Because the grading curve of the base material contains a defined filler fraction, the PU-RAP filler was also substituted proportionally.

#### 2.2.2. Design of the Test Variants

Within the scope of this work, three variants were worked out in addition to a reference variant, which is produced based on the initial material taken from [3]. The initial material is volumetrically substituted by the PU-RAP material in the individual grain fractions. To each variant a different proportion of PU-RAP material was added. This approach ensures that the same proportion of PU-RAP material is present in each grain fraction and that the grading curves defined in the previous study [3] can be realized. Table 2 shows the material composition.

The different variants are always made using the same procedure to ensure the greatest possible comparability. To produce the specimens for the investigation of the low-temperature behaviour and the fatigue testing, specimen plates measuring 32 × 26 × 4 cm${}^{3}$ were manufactured, from which the prismatic specimens are cut out. In order to avoid negative influence, the edge areas of the plates are removed.

The cylindrical specimens for the deformation resistance tests and the absorption capacity tests are prepared in single moulds with a diameter of 100 mm and a height of 60 mm.

**Figure 2:**
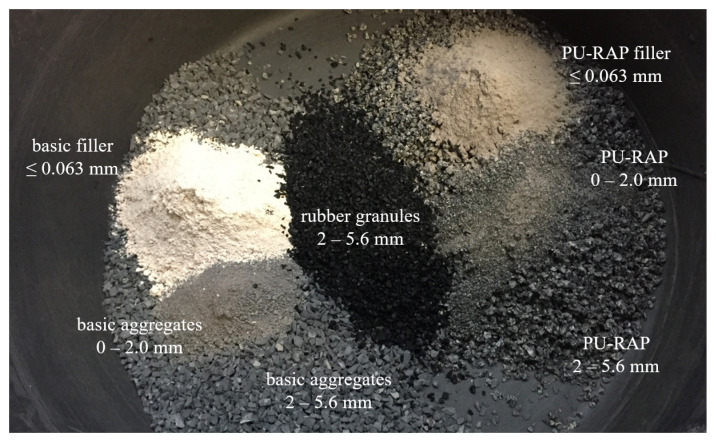
PU-RAP and basic material in visual comparison.

Looking visually at the PU-RAP compared to the original material, one difference in particular can be seen between the base filler and the recycled filler (see Figure 2). The lighter components are the basic materials and the more grey ones are the recycled ones.

All variants are made with 13 vol.% polyurethane and 7.5 vol.% rubber and the aggregates are partially substituted with PU-RAP at 25%, 50% and 75%.

Due to low laboratory capacities, only three variants (RAP0, RAP25, RAP50) were investigated in the low temperature and fatigue tests. Deformation resistance and absorption capacity were tested on all four variants (RAP0, RAP25, RAP50, RAP75).

When the mixture is fully coated by the binder during mixing, it is filled into the mould. Finally, the material is compacted by a hand roller. The mould filled with the PU-RAP mixture must rest and cure for at least 24 h. The asphalt plate can then be demoulded and sawed to the required specimen dimensions.

#### 2.2.3. Densities and Void Contents of Tested Specimens

The pycnometer method according to TP Asphalt-StB Part 5 [18] was used to obtain the maximum material densities for all materials as well as for the mixtures of the test variants. The asphalt mix is placed in a pycnometer and weighed. A defined amount of water is then added and the sample is vacuumed. In the process, all accessible air voids are eliminated. By determining the masses of the sample with and without water and taking into account the existing material densities, the material density of the pure asphalt mix can be determined. Subsequently, the bulk density of the finished specimens was determined according to TP Asphalt-StB Part 6 [19] due to immersion weighing. The void contents of the individual specimens can then be determined from the maximum densities and the bulk densities.

### 2.3. Selection of Suitable Test Methods

Test methods for bitumen-bound asphalt are used to investigate the material behaviour of the polyurethane-bonded asphalt, as these are used to evaluate its suitability for road use. These may not address the full material behaviour of the new material yet but they provide an initial opportunity of addressing the material and receiving a comparison to conventional asphalt.

In order to determine the overall material performance of the specimens with the added PU-RAP, the tests that were carried out in the previous study [3] are applied. Thus, a comparability of the results can be generated.

#### 2.3.1. Absorption Behaviour

As in [3], the measurement of the acoustic efficiency by the impedance measuring tube (AFD 1000-AcoustiTube ® according to *DIN EN ISO 10534-2* [20] is used in this study. The impedance measuring tube allows a simple and fast measurement of the absorption coefficient of materials. In this test, a long tube with a sonic rim contains a loudspeaker which acoustically excites the air enclosed in the tube. At the end of the tube, a cylindrical specimen is installed. The sound excitation causes the sound to propagate in the longitudinal direction of the tube and it hits the specimen, which reflects and/or absorbs the sound waves. A measuring device located behind the specimen measures the absorbed or reflected components of the sound waves. The method used in this study is called the wave separation method, which provides the absorption value α across a frequency range from 250 Hz to 2000 Hz [21].

#### 2.3.2. Deformation Resistance with Uniaxial Cyclic Compression Test (UCCT)

In the context of this work, the UCCT was chosen to test the deformation behaviour. Due to its haversine-impulse loading, it is suitable to represent the axle-load-simulating dynamic loading exerted on the pavement by passenger car and heavy-load traffic. The test is carried out in accordance with *TP Asphalt-StB, Part 25 B 1* [22]. The UCCT is used to determine the strain rate.

According to *TP Asphalt-StB, Part 25 B 1* [22], a cylindrical plane-parallel specimen is uniformly subjected to a haversine pulse-shaped pressure swell load. The test sequence is characterized by load cycles that cause axial deformation on the specimen. The deformation parameter of this test is the strain rate, which is used to determine the resistance to deformation. The specimen has to be prepared as a cylinder with a diameter of 100 ± 5 mm and a height of h = 60 ± 1 mm using drilled cores from laboratory plates. Contrary to the instructions in the regulations, the specimens in this study were manufactured using moulds with a diameter of 100 mm. During testing, the specimen is loaded by the haversine-pulse loading at an under stress of 0.025 MPa and a top stress of 0.35 MPa.

The deformation under the top load plate is recorded as a function of the loading cycle. The test is complete when either 10,000 load cycles are performed or a permanent deformation of more than 40‰ is reached.

#### 2.3.3. Fatigue Behaviour with Three Point Bending Test (3PB)

Pavement fatigue is generated by the application of repetitive loads. In general, the loads are significantly lower than the maximum bending tensile strength of the material and, due to their constant repetition, cause damage accumulation that ultimately results in the failure of the material. The failure then manifests itself in cracks, which typically start from the bottom of the layer and propagate up to the top [23].

In order to investigate the fatigue behaviour, the three-point bending test was used in this study. It is performed as a displacement-controlled test that describes the behaviour of a beam-shaped prismatic asphalt specimen. The test setup imitates the practical case in which a moving load axis passes over the asphalt pavement.

The decision to use the three-point bending test is due to the comparability of the test results, as it has been used to analyse the previous material compositions of Faßbender and Oeser [3] as well as the material of Renken [14].

The fatigue resistance of conventional asphalt is described by *DIN EN 12697-24 Asphalt-Test methods-Part 24: Resistance to fatigue* [24]. The specimens are subjected to a flexural load, which imitates rolling wheel loads. In detail, the prismatic specimens are tested at a temperature of 20 ${}^{\circ }$C ± 1 ${}^{\circ }$C. The test specimens, with a maximum grain size of 22 mm, can either be cut out of specially manufactured slabs or cut out of an existing pavement with a minimum thickness of 50 mm. Deviating from the specifications described in [24], the dimensions specified for PU asphalt test specimens in this study are 40 × 40 × 320 mm${}^{3}$ according to [3,14].

The specimen is installed in the test fixture for the three-point bending test and subjected to a sinusoidal load with constant amplitude by a centrally applied load application device until the stiffness drops to half its initial value. Because *DIN EN 12697-24* [24] only applies to the testing of conventional asphalt, the procedure according to [14] is used in this study, as in the investigation of the basic material [3]. In the first step, a static flexural strength test is carried out to determine the flexural strength of the material. The maximum deflection s_max_ received from this is the basis for the subsequent displacement-controlled cyclic loads, whose displacement amplitude results from 2/3 of the maximum deformation s_max_. The cyclically loaded specimens are then subjected to another static flexural tensile test to determine the residual flexural tensile strengths after dynamic loading.

#### 2.3.4. Low Temperature Behaviour

The service temperatures of pavements cover a wide temperature range which can significantly influence the material behaviour. Bitumen-bound asphalts, for example, are highly temperature-dependent and change their elastoplastic properties considerably with increasing or decreasing temperature. The temperature behaviour of polyurethane bound pavements has not been researched as much. In particular, the influence of the addition of PU-RAP has not been the subject of research so far. Therefore, in the following, the low-temperature behaviour is investigated with respect to the present variants of this study.

In addition to stresses due to traffic loads, cryogenic stresses in the pavement occur under the influence of cold temperatures. Superposition of these stresses can lead to damage if the tensile strength reserve of the pavement is not sufficient. For this reason, the study of the low-temperature behaviour of asphalt is of great importance. The low-temperature behaviour of asphalt can be described by means of direct tensile tests, cooling tests, relaxation tests and retardation tests. In this study, the focus is on the Uniaxial Tension Stress Test and the Thermal Stress Restrained Specimen Test, because they describe the low-temperature behaviour in a practical manner. The framework for this is *DIN EN 12697-46 Asphalt-Test methods for hot mix asphalt-Part 46: Resistance to cold cracking and low temperature behaviour in uniaxial tensile tests* [25]. The test procedure is analogous to the investigation of the low-temperature behaviour in [3]. The tested prismatic specimens with the dimensions 40 × 40 × 160 mm${}^{3}$ originate from specimen plates manufactured in the laboratory.

##### Uniaxial Tension Stress Test (UTST)

The aim of the UTST is to stretch the clamped specimen at a constant temperature until it fails. For this purpose, the prismatic specimen is firmly bonded with its two end faces in the test fixture and then subjected to a tensile load at a constant strain rate at a defined temperature level. In total, five temperature levels must be covered (+20 °C, +5 °C, −10 °C and −25 °C).

##### Thermal Stress Restrained Specimen Test (TSRST)

The TSRST differs from the direct tensile test only in the type of loading. In this test, the specimen is fixed again, the specimen length is kept constant, and a temperature induction follows, which causes temperature-induced stresses (cryogenic stresses) in the specimen. The test temperature is decreased from +20 °C by 10 K/h until the specimen fails or a minimum temperature of −40 °C is reached.

The difference between the tensile strength $\beta_{t}\left(T\right)$ and the cryogenic stress $\sigma_{cry}\left(T\right)$ is the tensile strength reserve.

## 3. Experimental Results and Discussion

### 3.1. Densities and Void Contents

**Figure 3:**
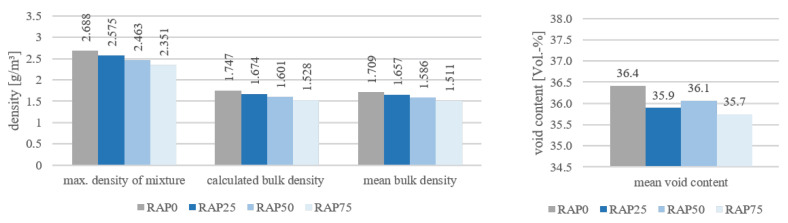
Mean maximum, calculated and experimentally determined bulk densities and void contents of all specimens.

Figure 3 shows the maximum densities, the calculated bulk densities and the experimentally determined bulk densities, as well as the void contents as averages for the variants.

First, it can be stated that the bulk densities of the variants which were previously determined by calculation are confirmed by experimental testing according to TP Asphalt Part 6 [19].

When considering the material properties in relation to the variants, a clear decreasing trend can be seen with regard to the densities. With increasing substituted PU-RAP, both the maximum material densities and the bulk densities decrease. This is due to the fact that by adding the PU-RAP, additional old binder as well as old rubber particles are present in the mixture, which lowers the total bulk density. The individual densities can be taken from Appendix A (Table A1).

The void content shows a decreasing trend. This reduction is not decisive because the bulk density was measured by dimensions according to [19], which is highly user dependent. Other methods have not yet been effective for open-pore materials.

### 3.2. Absorption Potential

The sound absorption coefficient is determined in accordance with *DIN EN ISO 10534-2* [20] in the impedance measuring tube.

The absorption coefficient can range from 0 to 1. The value 0 means that the surface reflects the sound completely. A value of 1, however, means that the sound is completely absorbed.

**Figure 4:**
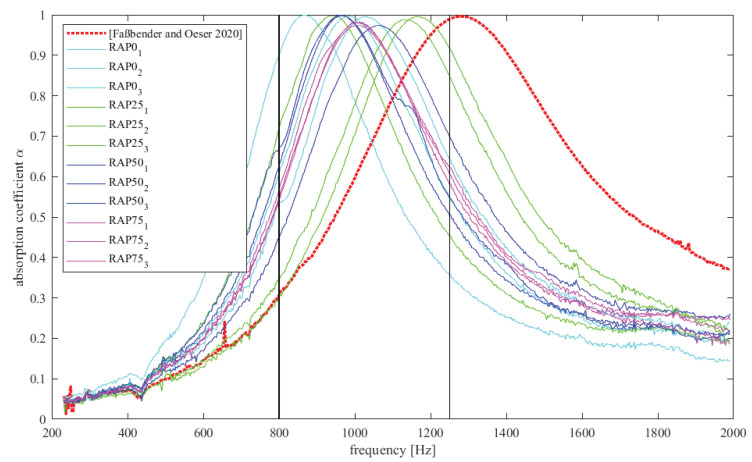
Absorption coefficient curves of the variants RAP0, RAP 25, RAP 50, RAP 75 and from Ref. [3].

Figure 4 shows the results of the measurements with the impedance measuring tube. The absorption coefficients α over the frequency are shown. The absorption curve of the previous study [3] is depicted with a red dotted line. All other curves are results of the current study. Shown here are all individual measurements of the different variants: (RAP0 (cyan), RAP25 (green), RAP50 (blue) and RAP75 (magenta)).

Through analysis of the results, it becomes apparent that all variants that were acoustically investigated exhibit distinctive absorption amplitudes with a maximum of almost α=1. This means that all samples have a significant absorption capacity in certain frequency ranges. From the literature [3,26,27], it is known that the degree of porosity influences the maximum absorption capacity. The higher the porosity, the higher the absorption coefficient. Considering these findings, it can be seen that the porosity of the variants presented here is generally very high, which is why a high absorption level is achieved in all tested variants.

In addition, the thickness of the layer as well as the resistivity are responsible for the frequency range in which the absorption curve is located. The thicker the layer, the lower the frequency range of the absorption amplitude. Furthermore, tortuosity affects the frequency range of the amplitude [26,27]. It can be seen that all variants, including the newly manufactured base variant RAP0 (cyan), exhibit an absorption curve in the lower frequency range compared to the variant from [3] (dotted red). This suggests that either the layer thickness or the tortuosity of the variants differ. Because the experimenters tried to make the layer thickness the same for all variants, the reason for the change of the frequency range is probably the tortuosity. This is presumably due to the fact that a two-component polyurethane was used here to produce the specimens instead of the one-component polyurethane used in [3]. If we omit the comparison with the variant from Oeser and Faßbender [3], variants RAP25, RAP50 and RAP75 show an increase in the frequency range compared with the reference variant, which may be due to an increase in tortuosity. The polyurethane wraps around the aggregates and does not collect in the gaps. Therefore, the channels in the layer could become narrower with a high void content present at the same time.

Looking at the samples tested in this study in Figure 4, it can be recognized that there is a scatter along the frequency range in the variants RAP0 (cyan), RAP25 (green) and RAP50 (magenta). Only the three test results of RAP75 (magenta) are stringent. In RAP25 and RAP75, in each case one measurement deviates slightly. It is found, however, that the curves move within the frequency range of 800 to 1250 Hz, which is an important frequency range for traffic noise according to [26] and can thus efficiently absorb and reduce traffic noise for human hearing [3].

Compared to the previous study [3], the results show that the proportion of addition of the PU-RAP does not reveal any apparent tendency about improvement or deterioration of the absorption properties. Moreover, it seems that the addition of a two-component polyurethane binder has a positive effect on the frequency range by moving the curves into the lower frequency range.

In [3], an effective normalized absorption value was introduced for evaluation, which reflects the absorption power in the decisive frequency range between 800 and 1250 Hz. This value is the area under the curve in the interval between 800 and 1250 Hz related to the difference between 800 and 1250 Hz. It takes into account not only the height of the absorption curve, i.e., the maximum absorption capacity, but also the width of the amplitude.

**Figure 5:**
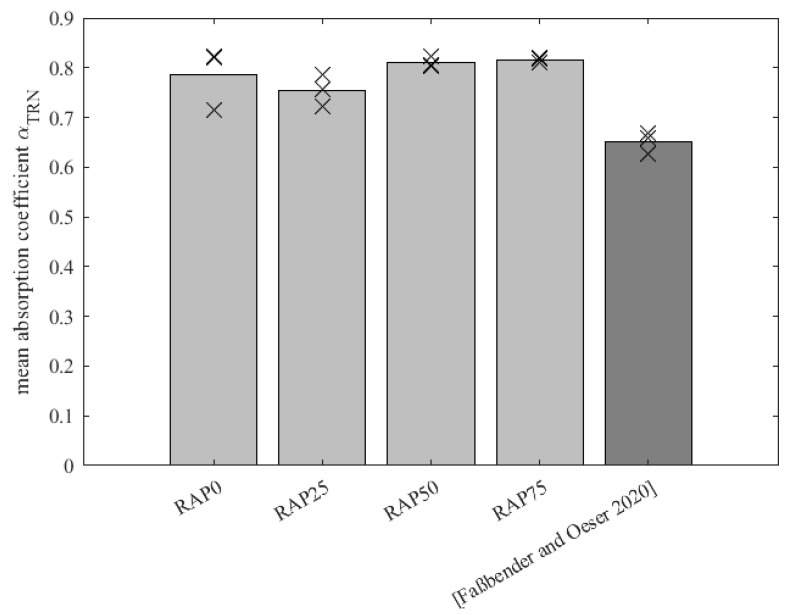
Mean absorption coefficient values of the variants RAP0, RAP25, RAP50, RAP 75 and Ref. [3].

The absorption values are shown in Figure 5 as mean values in the form of bars. The markers show the individual results. As already indicated in Figure 4, it can be seen that the absorption capacity has increased, which could be due to the new composition with PU-RAP compared to the variant from [3]. Furthermore, it is noticed that the addition of the PU-RAP shows a slight positive trend, indicating that a higher absorption level is achieved with more PU-RAP added. However, this is a vague statement when considering the individual values.

In principle, it can be concluded that the absorption capacity of the variants made of recycled material is better compared to the original as their absorption level is higher. An increased percentage of polyurethane (old and fresh polyurethane) may support the efficiency of the absorption. As a result, the material can be used with confidence and will continue to perform effectively in terms of acoustic efficiency.

### 3.3. Uniaxial Cyclic Compression Test

During the cyclic compression test according to [22], a cylindrical specimen is subjected to repetitive compressive loading with load pauses. The cyclic loading is used to investigate the resistance to deformation of the tested material.

As a result, the strain of the specimen is obtained which makes it possible to predict the extent to which the material will deform in the long term. The strain is determined according to [22].

**Figure 6:**
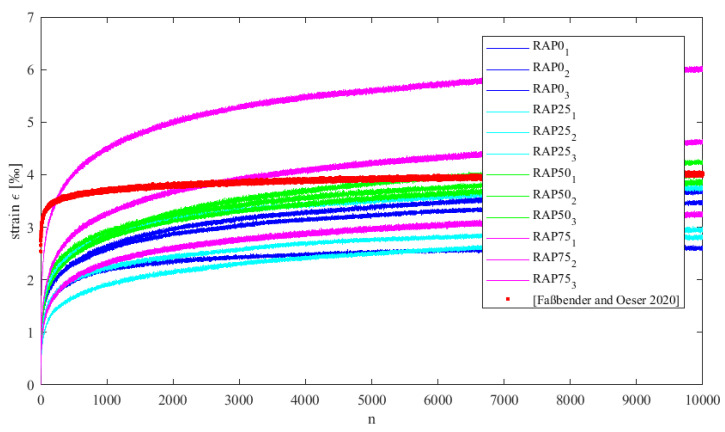
Strain of variants RAP0, RAP25, RAP50, RAP75 and mean strain of Ref. [3] due to the uniaxial cyclic compression test.

The deformation curves in Figure 6 describe the accumulated strains due to load application. As can be seen in Figure 6, the deformation curves of the investigated specimens lie in a strain range between 0 and about 6 ‰. It can be seen that variant RAP75 (75% PU-RAP) exhibits a more pronounced deformation behaviour, which is visible due to a stronger scatter of the individual results. All deformation curves follow an asymptotic trend along the entire testing period of 10,000 load cycles. No inflection point is visible in the deformation curve, which is usual for conventional asphalt. This fact indicates that no sudden failure of the specimens occurs during the loading process.

In principle, it can be concluded that the specimens undergo an irreversible change in shape as a result of the input energy, although this change is quite small during long-term loading. This indicates that there is a high deformation resistance of the material. Nevertheless, variant RAP75 yields larger deviations. The deviations could possibly be due to the rubber content contained in the PU-RAP as well as in the base compound.

**Figure 7:**
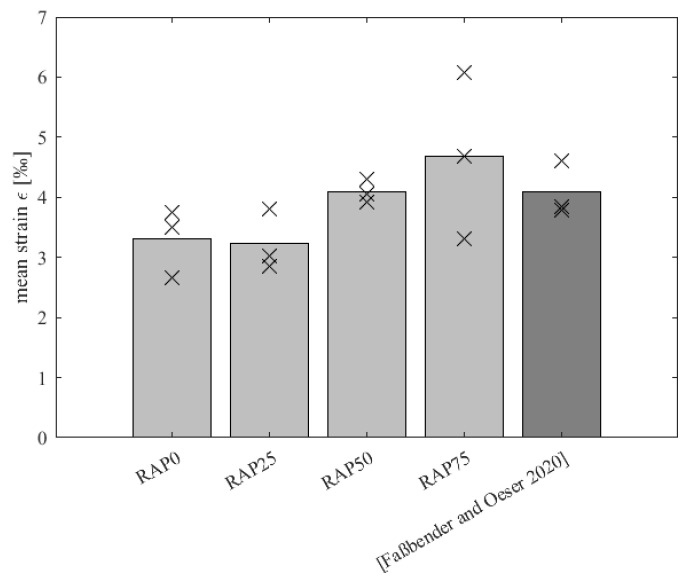
Mean maximum strain of variants RAP0, RAP25, RAP50, RAP75 and Ref. [3] at 10,000 load cycles.

An illustration of the mean maximum strains at a load cycle number of n = 10,000 is shown in Figure 7. Looking at the bar chart in detail, it can be seen that no clear trend of the PU-RAP addition is visible. Initially, it seems as if there would be a deterioration of the deformation resistance with increasing PU-RAP, because the bars of the variants RAP25, RAP50 and RAP75 show a positive trend. When considering the individual values, that are shown here as crosses, a scattering of the results is noticeable, which cannot surely confirm the trend. However, no exact statement about a clear trend of the PU-RAP addition is possible.

A comparison with the variant from [3] (red line in Figure 7), in which the one-component polyurethane binder Elastan 6568/103 was used, shows that the variants from this study present ultimate strains of the same order of magnitude and thus have an equivalent high potential of deformation resistance. Obviously, the deformation resistance curve differs from the RAP variants, because it shows a rapid and short-term increase in strain, which is much steadier over the duration of the test.

### 3.4. Three Point Bending Test

The three-point bending test (3PB) with the modifications according to [14] is carried out to assess the fatigue behaviour of the specimens produced with PU-RAP. The procedure is such that first a static flexural tensile test is carried out to determine the static flexural tensile strength, followed by a cyclic sinusoidal fatigue load with 20,000 load cycles. Finally, the residual bending tensile strength is determined by means of another static bending tensile test in accordance with *DIN EN 12390-5* [14,28] in order to assess the remaining material resistance.

**Figure 8:**
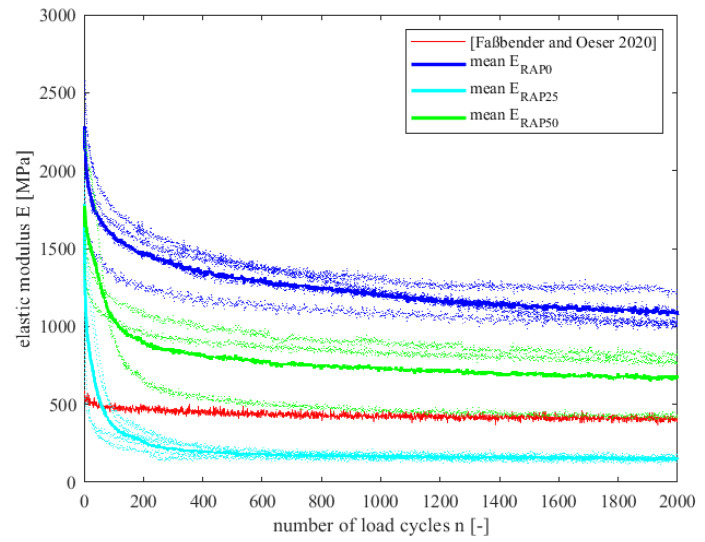
Course of the diminishing e-modulus of variants RAP0, RAP25, RAP50 and [3].

Figure 8 presents the course of the diminishing e-modulus for the tested specimens during loading. Outliers were eliminated beforehand. All curves are assigned to the respective variant by colour. It can be seen that the curves of variant RAP50 lie in a narrow corridor. The curves of the variants RAP0 and RAP25 each have a curve that deviates slightly more in its course. The highlighted curves indicate the average curve of the e-modulus of the respective variants RAP0, RAP25 and RAP50. Although constant test conditions were maintained both in the manufacturing process and in the testing process, there is a large spread within the course of the e-modulus curves. A correlation between the proportional addition of the PU-RAP and the present Young’s modulus curves is not evident here.

A comparison of the fatigue curves of this study with the previous study according to [3] clearly shows that the material behaviour has changed considerably. The curves of the variants RAP0, RAP25 and RAP50 start at a high Young’s modulus and decrease exponentially compared to the curves of [3], which are rather constant and almost identical in comparison. In Figure 8, it is not possible to see that three variants are shown, because they run superimposed.

The variants of this study were produced with two-component polyurethane, contrary to the variant from [3]. Because even the reference variant RAP0, which only has the different binder, shows such a strong development here, it becomes clear that the used binder has an enormous influence on the fatigue resistance.

According to *DIN EN 12697-24* [24], the conventional failure criterion is used to evaluate the fatigue behaviour. After the test is done, the stiffness is calculated across the number of load cycles with Equation (1).

(1) $$\begin{array}{c} \left|E\right|=\frac{K_{a}\cdot l^{3}}{4\cdot w_{a}\cdot b\cdot h^{3}}\\ absolute\;elastic\;modulus\;[MPa]=|E|\\ force\;amplitude\;\left[N\right]=K_{a}\\ support\;span\;[mm]=l\\ maximum\;deflection\;of\;the\;center\;of\;the\;beam\;\left[mm\right]=w_{a}\\ width\;of\;the\;specimen\;[mm]=b\\ height\;of\;the\;specimen\;[mm]=h \end{array}$$

A specimen is considered fatigued after the number of load cycles at which the initial stiffness ($E_{100}$) has decreased by half. The initial stiffness is defined as the stiffness of the specimen that exists after the 100th load change. The test is completed after 20,000 load cycles.

Table 3 shows the fatigue test results with respect to the fatigue criterion. For this purpose, the initial stiffnesses $E_{100}$, the load cycles when the fatigue criterion n is reached and the stiffness at the end of the test $E_{20,000}$ are listed for all tested specimens.

Based on the values from Table 3, it can be confirmed that the reference variant RAP0 has good fatigue resistance. This, similar to the variant of [3], does not reach the failure criterion, except in one sample. Compared to the variant from [3], the elasticity curves in this study are at a much higher level. The fact that in this study the two-component binder was used instead of the one-component one could be a reason. In previous investigations, it was found that the one-component polyurethane is more elastic and thus has lower elastic moduli. The fatigue criterion shows that the RAP0 and RAP50 variants have high fatigue resistance except for one specimen each. Apart from the exceptions, they do not reach the fatigue criterion. The fatigue curves from Figure 8 also show that the RAP0 and RAP50 variants have a higher initial stiffness, which can be seen in the position of the curves. Variant RAP25, on the other hand, reaches the failure criterion after a very short number of load cycles under 202 load cycles for every sample tested, which is about 1% of the total number of load cycles. The lower level of Young’s moduli is also reflected in the fatigue curves of variant RAP25.

A direct influence of the addition of PU-RAP cannot be detected in this case. The positioning of the RAP0 and RAP50 variants could indicate that the PU-RAP possibly lowers the Young’s modulus, although no clear trend can be defined here. However, this could be explained by the fact that the higher the PU-RAP, the higher the rubber and polyurethane content in the compound, which is why the e-modulus is lower. However, variant RAP25 unfortunately does not support this statement. Nevertheless, both the RAP0 and RAP50 variants show that they have a high fatigue resistance, because they do not reach the fatigue criterion. As a conclusion, variant RAP25 must be retested. The trend of the RAP0 and RAP50 variants makes sense in principle. Maybe an unfavourable PU-RAP charge was selected during specimen fabrication, resulting in a useless plate from which the specimens were cut out.

**Figure 9:**
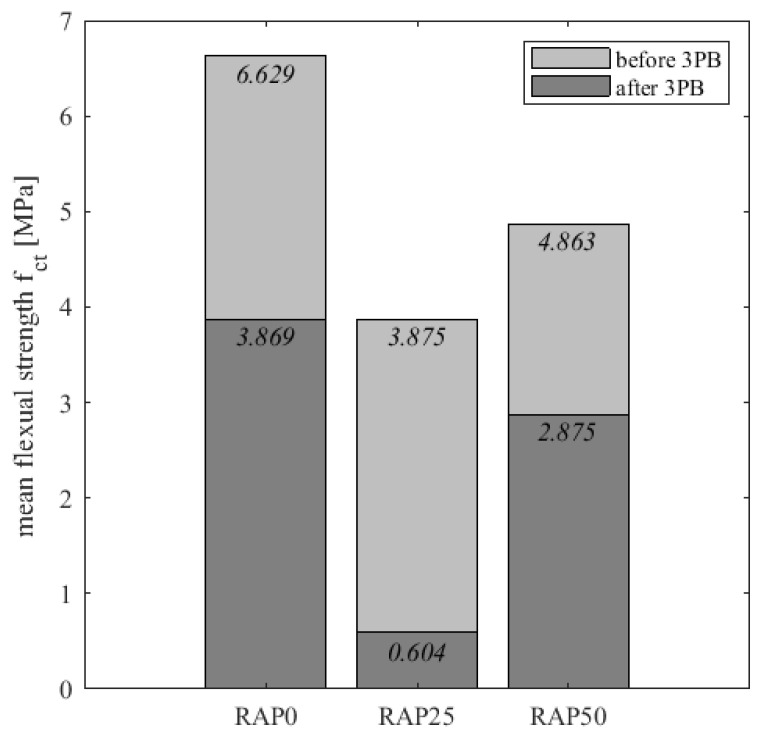
Flexural strength comparison before and after 3-point bending test.

Looking at the results of the flexural strength tests before and after the fatigue test, it can be seen that flexural loading caused extensive damage to the microstructure. The values before and after fatigue show this effect very clearly (Figure 9). Thus, the flexural strength of variant RAP0 has decreased by 42%, the flexural strength of RAP25 has decreased by 84% and the flexural strength of variant RAP50 has decreased by 41%. Here, too, variants RAP0 and RAP50 exhibit higher flexural tensile strengths than variant RAP25, where the flexural tensile strength is already strikingly low at the beginning. The high damage potential of 84% again indicates that there is a structural problem with variant RAP25 that needs to be investigated again. However, variants RAP0 and RAP50, with about 40% reduction in flexural tensile strength, also clearly indicate the damaging effect of fatigue testing. An influence of the PU-RAP on the fatigue resistance cannot be detected here.

### 3.5. Results of Low-Temperature Behaviour

To analyse the low temperature behaviour of polyurethane bound pavements with substituted PU-RAP, the procedure according to [25] is applied.

Both the Tension Stress Test (UTST) and the Thermal Stress Restrained Specimen Test (TSRST) are used to test the low-temperature behaviour. The UTST gives tensile strengths at the temperature levels 20 °C, 5 °C, −10 °C and −25 °C, which are represented by a cubic spline function. The TSRST indicates the stresses that develop in an expansion restrained specimen when it is cooled down. From the two test results, it can be determined how high the stress bearing capacity of the pavement is and how much of this is available as a reserve for further loads, e.g., from traffic. This reserve is called tensile strength reserve.

**Figure 10:**
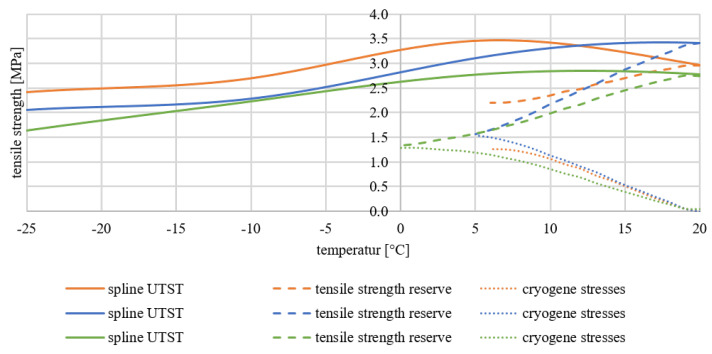
Results of the UTST and TSRST: RAP0 (orange), RAP25 (blue), RAP50 (green).

Figure 10 shows the results of the UTST and TSRST and the resulting tensile strength reserves of the variants RAP0, RAP25 and RAP50.

In Figure 10, it was recognized that in all TSRST of the variants RAP0 (orange), RAP25 (blue) and RAP50 (green), the resulting cryogenic stresses lead to early material failure. The samples were already damaged at temperatures starting at about 5 °C (RAP0 and RAP25). Variant RAP50 broke at a temperature of 0 °C. Thus, a tensile strength reserve is found here for all variants only in the temperature range between 5 °C or 0 °C and 20 °C.

Considering the tensile strength reserves of the different variants, no clear influence of the different addition of the PU-RAP can be determined. Variant RAP0 starts at 20 °C at a tensile stress of 2.9 MPa and decreases to about 2.2 MPa at 6 °C. Variant RAP25 starts at 3.4 at 20 °C and runs towards 1.6 MPa at 5 °C, and variant RAP50 starts at 2.7 (20 °C) and continues to 1.3 (0 °C). It is true that the RAP0 and RAP25 variants, which contain a lower PU-RAP content, have a higher tensile strength reserve. However, variant RAP50 covers a broader temperature range and contains the highest proportion of PU-RAP. Thus, it is assumed that the addition and the proportion of PU-RAP does not have a significant effect on the low-temperature behaviour.

**Figure 11:**
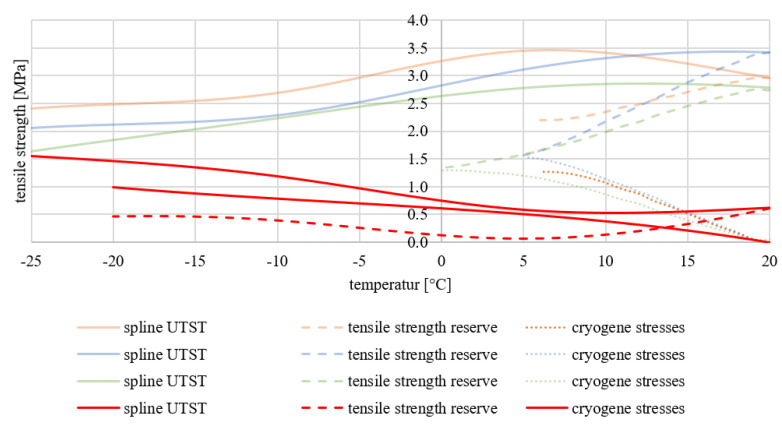
Results of the UTST and TSRST compared to [3]: RAP0 (orange), RAP25 (blue) and RAP50 (green), Ref. [3] (red).

If this is compared with the low-temperature behaviour from [3] (black curves), it can be seen directly in Figure 11 that the materials differ greatly in their low-temperature behaviour. The big difference between the two mixtures from [3] and from this study can be found in the different binders that are used. The two-component polyurethane used here is very sensitive to low temperatures. This trend was already observed in the research project INNO-PAVE [13] when it came to selecting the optimum binder. However, that such a huge difference is found in the context of this study was not expected.

Figure 11 shows that the cryogenic stresses in the TSRST do not damage the specimens of [3] until a temperature of −20 °C is reached. Previously, they rose steadily with decreasing temperature but withstood the low temperatures. The tensile strength in [3] is quite low. This is due to the low quantity of binder used there. In the final mixture, the binder content was therefore adjusted.

Finally, the influence of the added PU-RAP does not seem evident in terms of the low-temperature behaviour.

## 4. Conclusions

For the reuse of reclaimed asphalt, it is standard practice in Germany to add RAP to the new asphalt mixture in order to reduce waste and and to conserve and reuse energy intensive resources. This approach is not only important in bitumen-bound asphalts. This strategy should also be continued with regard to innovative and sustainable pavements as the action plans from the EU and UN demand. Therefore, this study investigated whether the addition of PU-RAP has an effect on the material performance of the polyurethane-bound rubber-modified pavement. Test methods for bituminous asphalt were used for this purpose and modified where necessary. In general, this study has shown that the addition of PU-RAP has an influence on the material behaviour, which, however, does not show a clear trend. However, this study has shown in detail, that:

- The addition of PU-RAP has a positive influence on the absorption capacity of the polyurethane-bound rubber-modified pavement, as the frequency range can be adjusted.
- The addition of PU-RAP has no significant effect on the deformation behaviour of polyurethane-bound rubber-modified pavement.
- The addition of PU-RAP does not affect the resistance to fatigue of the polyurethane-bound rubber-modified pavement.
- The addition of PU-RAP has no effect on the low-temperature behaviour of the polyurethane-bound rubber-modified pavement.

It can therefore be stated very clearly that no damaging effect has been caused by the addition of PU-RAP and so it can be assumed with certainty that PU-RAP can be added to fresh mixtures free of any problems and without affecting the material performance. In addition, due to the deviation to an alternative binder, a statement could be made about the effect of the two-component binder, which greatly affects the performance of the material and worsens the performance compared to the variant from Faßbender and Oeser [3].

In order to be able to evaluate the study holistically and conclusively, the following points must be considered in further studies in detail:

- The RAP was freshly made, unstressed and unaged when it was reconditioned. The study, in the first step, intended to show the feasibility of reusing the PU-RAP. In the next step, it makes sense to take the RAP material from already stressed pavements and to investigate the functionality.
- All tests should be prepared and tested again with the one-component binder from [3] to generate comparability. The influence of the PU-RAP can likely be worked out even better in this case.
- Additional specimens should be tested to support the conclusions made in this study.

## Figures and Tables

**Table 1:** Mix design.

Mixture Components	Amount	Unit
aggregates	80.5	vol.%
rubber granules	6.5	vol.%
two-component polyurethane	13	vol.%

**Table 2:** Substituted PU-RAP proportion of the aggregates fraction.

Variant		RAP0	RAP25	RAP50	RAP75
PU-RAP	[vol.%]	0	25	50	75

**Table 3:** Results of fatigue testing.

Variant	Specimen No.	$E_{100}$ [MPa]	$n(0.5\cdot E_{100})$ [-]	$E_{20,000}$ [MPa]
[3]	-	537	not reached	386
RAP0	5	1671	not reached	1013
	7	1998	not reached	1212
	8	1903	not reached	1101
	9	2232	1262	1042
RAP25	7	467	202	152
	8	1431	62	173
	9	1393	50	137
	10	558	53	133
RAP50	5	2091	82	430
	7	1460	not reached	831
	10	1266	not reached	779

## Data Availability

Not applicable.

## Abbreviations

The following abbreviations are used in this manuscript:

3PB	three point bending beam test
PU	polyurethane
PU-RAP	polyurethane based reclaimed asphalt pavement
RAP	Reclaimed asphalt pavement
TSRST	Thermal Stress Restrained Specimen Test
UTST	Tension Stress Test

## Appendix A

The following Table A1 shows the basic aggregate sizes and the related maximum densities of the additives of the designed mix.

**Table A1:** Aggregate sizes and maximum densities of the designed mix.

	Aggregate Size [mm]	Maximum Density [g/cm${}^{3}$]
lime sandstone filler	≤0.063	2.730
PU-RAP filler	≤0.063	2.582
basalt stone	0.063–2.0	3.050
PU-RAP	0.063–2.0	2.588
basalt stone	2.0–5.6	3.050
PU-RAP	2.0–5.6	2.605
rubber super-grob	2.0–5.6	1.100
polyurethane	-	1.100

The following Table A2 shows the typical physical properties of the polyurethane Elastopave^®^ 6551/102 from BASF Polyurethanes GmbH [10].

**Table A2:** Typical physical properties.

Characteristics	Unit	Measured Value	Method
Hardness	Shore ID	72	DIN 53505
Tensile strenth	N/mm²	32	DIN EN ISO 527
Elongation	%	40	DIN EN ISO 527
Tear strength	N/mm	40	DIN 53515
Density	g/cm³	1.1	DIN 53420
